# A Case of Severe Hypercalcemia in a Patient Taking Tirzepatide: Potential Risk Factors

**DOI:** 10.7759/cureus.113391

**Published:** 2026-07-26

**Authors:** Safiah Iqbal, Faiza Javed, Samson O Oyibo

**Affiliations:** 1 General Medicine, Peterborough City Hospital, Peterborough, GBR; 2 Acute Medicine, Peterborough City Hospital, Peterborough, GBR; 3 Diabetes and Endocrinology, Peterborough City Hospital, Peterborough, GBR

**Keywords:** gip and glp-1 receptor agonist, non-parathyroid hypercalcemia, risk-factors, severe hypercalcemia, tirzepatide or mounjaro

## Abstract

Hypercalcemia is commonly caused by hyperparathyroidism and malignancy. Medication-induced hypercalcemia is less common. Tirzepatide is a dual glucagon-like peptide-1/glucose-dependent insulinotropic polypeptide receptor agonist (GLP-1/GIP RA) used to aid weight loss and glycemic control. It is associated with gastrointestinal side effects, but its effect on calcium homeostasis has not been well reported. We report the case of a 75-year-old woman with a history of type 2 diabetes, hypertension, obesity, chronic kidney disease, and post-surgical hypothyroidism and hypoparathyroidism, who presented with severe symptomatic hypercalcemia one month after starting tirzepatide while taking bendroflumethiazide, calcium carbonate, and alfacalcidol. Her serum calcium levels had been in the normal treatment range before the initiation of tirzepatide. All other possible causes of hypercalcemia were ruled out. Her calcium levels returned to normal after intravenous fluid therapy and withholding tirzepatide, bendroflumethiazide, and calcium carbonate. This case highlights the potential occurrence of severe hypercalcemia during the concurrent use of tirzepatide with bendroflumethiazide, a calcium supplement, and vitamin D in the context of chronic kidney disease. With the increasing use of tirzepatide, healthcare workers need to be mindful of the interaction between tirzepatide and these potential risk factors. While further research is required, we suggest serum calcium monitoring during similar clinical scenarios.

## Introduction

Obesity and type 2 diabetes mellitus have significant public health consequences. As of 2023, 650 million people were classified as obese, and diabetes affects 537 million people worldwide. These figures are expected to continue to increase [1]. Tirzepatide is a dual glucagon-like peptide-1 (GLP-1)/glucose-dependent insulinotropic polypeptide (GIP) receptor agonist (GLP-1/GIP RA) approved for improving glycemic control in patients with type 2 diabetes mellitus and for chronic weight management in patients with obesity. GLP-1 and GIP are both intestinal incretins that stimulate the brain satiety center and pancreatic insulin release during mealtimes. GLP-1 also slows gastric emptying. The net effect is to reduce food intake and improve metabolism [1,2]. In a major study of 140,308 patients with type 2 diabetes, tirzepatide was found to be associated with lower hazards of all-cause mortality, adverse cardiovascular events, acute kidney injury, and adverse kidney events compared with GLP-1 RA [3]. The common side effects associated with GLP-1/GIP RAs are gastrointestinal effects, of which nausea and diarrhea are most frequent. Rarely, it can cause severe hypoglycemia, acute pancreatitis, cholelithiasis, and cholecystitis [4].

Normal serum calcium levels range from 2.2 to 2.6 mmol/L. Calcium homeostasis is maintained by three processes: intestinal absorption, bone turnover, and renal reabsorption [5]. These processes are regulated by parathyroid hormone (PTH), calcitriol, and serum ionized calcium and their receptors, namely, PTH receptor, vitamin D receptor, and the calcium-sensing receptor, respectively [6]. Hypercalcemia is commonly caused by PTH excess (primary hyperparathyroidism) due to parathyroid gland adenoma (80% of cases) or hyperplasia (10-15%) and malignancy, together accounting for 80-90% of hypercalcemia cases [7]. Other causes include thyrotoxicosis, acute adrenal insufficiency, excessive intake of dietary calcium, granulomatous disease, medications (including thiazide diuretics, lithium, theophylline), and vitamin D toxicity [5]. Severe dehydration can cause severe hypercalcemia, but this is rare [8]. Although hypercalcemia is generally an incidental finding and usually asymptomatic, multiple symptoms can occur, including thirst, polyuria, nausea, vomiting, loss of appetite, abdominal pain, bone pain, fatigue, confusion, depression, and palpitations [9].

Thiazide diuretics inhibit the sodium-chloride co-transporter (NCC) in the distal convoluted tubule, resulting in natriuresis, diuresis, and reduced intracellular sodium. This, in turn, increases the activity of the sodium-calcium exchanger (NCX), which pushes calcium out of the cell in exchange for sodium, causing increased calcium reabsorption into the blood, reducing urinary calcium excretion which, in rare instances, have resulted in mild hypercalcemia [10,11].

The association between tirzepatide use and developing hypercalcemia is not well reported. We highlight a case of severe hypercalcemia following the introduction of tirzepatide in a patient taking a thiazide diuretic, a calcium supplement, and a vitamin D supplement in the context of chronic kidney disease. This incident was appropriately reported to the Medicines and Healthcare products Regulatory Agency (MHRA), United Kingdom (reference number: GB-MHRA-MED-202603101036032780-MVNFD).

## Case presentation

Medical history and demographics

A 75-year-old female patient presented with a four-week history of altered mental status, poor memory, fatigue, thirst, and generalized weakness. Symptoms developed approximately one month after the initiation of tirzepatide 2.5 mg subcutaneously weekly. She denied any abdominal pain, constipation, or polyuria. Her medical history included type 2 diabetes, hypertension, obesity, chronic kidney disease, hypothyroidism, and hypocalcemia secondary to hypoparathyroidism. Hypothyroidism and hypoparathyroidism occurred after a total thyroidectomy and parathyroidectomy for a large multinodular goiter and primary hyperparathyroidism, respectively, two years prior to presentation. Her medication list included calcium carbonate 1.25 g daily, alfacalcidol 1 mcg daily, bendroflumethiazide 2.5 mg daily, tirzepatide 2.5 mg subcutaneously once weekly, atorvastatin 40 mg daily, levothyroxine 100 mcg daily, omeprazole 20 mg daily, and amitriptyline 10 mg daily. She denied any over-the-counter herbal remedies, dietary supplements, or alternative therapies. She also denied the use of tobacco, alcohol, or recreational drugs. She was independent with all activities of daily living. She had no significant family history.

On examination, she was confused and lethargic but able to obey simple commands. She had a respiratory rate of 17 breaths per minute, blood oxygen saturation levels of 96% on room air, blood pressure of 136/79 mmHg, heart rate of 74 beats per minute, and a temperature of 36.7 degrees Celsius. Chest, abdominal, and neurological examination did not reveal any abnormal findings. She weighed 78 kg (body mass index: 35.2 kg/m^2^).

Investigations

Initial blood results demonstrated severe hypercalcemia, low vitamin D levels, mild hypokalemia, mild hypomagnesemia, suppressed PTH levels in keeping with underlying hypoparathyroidism, and stage 2 acute kidney injury (Table 1). Further investigations (full blood count, liver enzymes, serum protein electrophoresis, serum angiotensin-converting enzyme [ACE], C-reactive protein, erythrocyte sedimentation rate) were normal. An electrocardiograph demonstrated sinus rhythm and left ventricular hypertrophy. Historical results indicated that her baseline calcium levels ranged from 1.91 to 2.26 mmol/L from after her parathyroid surgery two years prior up until two months before tirzepatide was initiated. Her baseline estimated glomerular filtration rate (eGFR) was 30-40 mL/min/1.73 m^2^.

**Table 1:** Blood results on admission eGFR, estimated glomerular filtration rate

Laboratory test	Result	Normal range
Hemoglobin	121	115-165 g/L
White cell count	9.1	4.0-11.0 x 10^9^/L
Platelets	219	150-400 x 10^9^/L
Sodium	138	140-145 mmol/L
Potassium	3.0	3.5-5.3 mmol/L
Chloride	99	95-108 mmol/L
Creatinine	251	45-84 µmol/L
Urea	11.6	2.5-7.8 mmol/L
eGFR	16	>60 mL/min/1.73 m^2^
Corrected calcium	4.27	2.2-2.6 mmol/L
Magnesium	0.62	0.7-1.0 mmol/L
Parathyroid hormone	<0.4	0.8-1.5 pmol/L
Phosphate	0.87	0.8-1.5 mmol/L
Total vitamin D	19	>50 nmol/L
Free thyroxine	27.5	11.9-21.6 pmol/L
Thyroid stimulating hormone	1.5	0.3-4.2 mU/L
Glycated hemoglobin (HbA1c)	56	<42 mmol/mol

Treatment

Bendroflumethiazide, calcium carbonate, and tirzepatide were all stopped during her admission. She was administered intravenous fluids for rehydration, along with oral magnesium and potassium replacement therapy.

Outcome and follow-up

Serum calcium levels normalized, and her eGFR improved to 25 mL/min/1.73 m^2^ by day 8 of admission. Following discharge, bendroflumethiazide and calcium carbonate were not restarted. Tirzepatide was restarted at a dose of 2.5 mg once weekly, alfacalcidol 1 mcg daily was continued, and cholecalciferol (vitamin D3) 800 units daily was added. At her one-month and two-month follow-ups, serum calcium levels remained in the acceptable range of 2.1-2.3 mmol/L, and kidney function returned to baseline (30-40 mL/min/1.73 m^2^). The patient reported complete resolution of symptoms and returned to her baseline functional status.

## Discussion

We report a case of severe hypercalcemia occurring one month after the initiation of tirzepatide in a patient with chronic kidney disease who was also taking bendroflumethiazide, low-dose calcium carbonate, and alfacalcidol. Despite taking these medications concurrently for two years, the patient’s serum calcium levels had always been in the treatment range until the initiation of tirzepatide.

Primary and secondary hyperparathyroidism were ruled out by a low PTH level, which was consistent with previous post-surgical hypoparathyroidism. Hypervitaminosis D was ruled out by low serum vitamin D levels. Multiple myeloma was ruled out by a normal serum protein electrophoresis and immunoglobulin level. Inflammatory and granulomatous diseases were ruled out by normal inflammatory markers and serum ACE. There was no evidence of levothyroxine overdosing, and the patient was not taking lithium. The patient denied excessive ingestion of calcium and vitamin D supplements, and she drank adequate amounts of fluid. Urinary calcium-creatinine ratio was not performed, abd thus it was not possible to identify any bendroflumethiazide-related reduction in urinary calcium excretion. However, the patient had been on bendroflumethiazide for two years after parathyroidectomy without any hypercalcemic episode.

An extensive literature review did not reveal any case of severe hypercalcemia directly related to GLP-1/GIP RAs or GLP-1 RAs used as a sole medication or monotherapy. However, a search did reveal four case reports of severe hypercalcemia in patients taking a GLP-1/GIP RA or GLP-1 RA with other medication, such as thiazide diuretics, calcium, and vitamin D supplements [12-15]. These cases shared common themes (Table 2). All the patients were above the age of 50 years, three had type 2 diabetes, one had pre-diabetes, three had hypertension, two had chronic kidney disease, two were taking a thiazide diuretic, three were taking a calcium supplement, two were taking a vitamin D supplement, and all of them had started tirzepatide or a GLP-1 RAs within three months of presenting with severe hypercalcemia. Additionally, they all had stable normal calcium levels before starting tirzepatide or a GLP-1 RA [12-15].

**Table 2:** Published reports of hypercalcemia occurring in patients taking a GLP-1/GIP or a GLP-1 receptor agonist GLP-1, glucagon-like peptide-1; GIP, glucose-dependent insulinotropic polypeptide

Study	Age and gender of the patient	Concurrent medical history	Concurrent medication	Serum calcium at presentation	Period of commencing GLP-1/GIP or GLP-1 receptor agonist prior to presentation	Action taken in addition to standard therapy resulting in rapid resolution
Patel et al., 2021 [12]	60-year-old male	Type 2 diabetes, obesity, controlled multiple myeloma, chronic kidney disease	Liraglutide, calcitriol, calcium carbonate	3.24 mmol/L	1 month	Liraglutide, calcium carbonate and calcitriol stopped
Nduma et al., 2025 [13]	65-year-old female	Type 2 diabetes, hypertension, hypothyroidism, chronic kidney disease	Tirzepatide, losartan, hydrochlorothiazide	4.48 mmol/L	3 days	Tirzepatide and hydrochlorothiazide stopped
Nguyen et al., 2026 [14]	70-year-old female	Pre-diabetes, obesity, obstructive sleep apnea, osteoporosis, hypertension, hyperlipidemia	Tirzepatide, amlodipine, losartan, atorvastatin, aspirin, calcium supplement, cholecalciferol (vitamin D3)	3.27 mmol/L	3 months	Tirzepatide, calcium supplements, and vitamin D stopped
Dushime and Ahmad, 2026 [15]	54-year-old female	Type 2 diabetes, hypertension, obesity	Semaglutide, losartan, amlodipine, calcium supplement, hydrochlorothiazide	4.12 mmol/L	2.5 months	Calcium supplement and hydrochlorothiazide stopped

The mechanism by which GLP-1/GIP RAs and GLP-1 RAs can directly or indirectly affect calcium homeostasis and result in hypercalcemia is unknown. Mechanisms proposed by the authors of the four case reports include the following: (1) GLP-1 RA-induced slowing of bowel transit time and gastric emptying may allow for increased absorption of calcium supplements and co-ingested cholecalciferol, calcitriol or alfacalcidol, (2) thiazide diuretics enhance renal reabsorption of calcium and can cause mild hypocalciuric hypercalcemia and thus when taken with GLP-1 RAs may potentially cause severe hypercalcemia, and (3) the reduced renal clearance in chronic kidney disease may just exacerbate the first two proposed mechanisms of action, especially when GLP-1 RAs, calcium or vitamin D supplements, and thiazide diuretics are taken concurrently [12-15].

Based on the above published reports and our case report, the following can be potential risk factors for the development of hypercalcemia in patients taking GLP-1/GIP RAs or GLP-1 RAs: (1) concurrent intake of calcium or vitamin D supplements, (2) concurrent intake of a thiazide diuretic, and (3) the presence of chronic kidney disease. Continued reporting of similar cases is important for validation of these potential risk factors.

## Conclusions

This case highlights a potential synergistic effect of the concurrent administration of tirzepatide, a thiazide diuretic, a calcium supplement, and vitamin D in a patient with chronic kidney disease. Given the temporal relationship observed and the increasing use of GLP-1/GIP RAs and GLP-1 RAs, further investigation is required to assess their effect on calcium homeostasis in the presence of the above-mentioned potential risk factors. We suggest calcium monitoring in these clinical scenarios.
